# A prospective, mixed-methods, before and after study to identify the evidence base for the core components of an effective Paediatric Early Warning System and the development of an implementation package containing those core recommendations for use in the UK: Paediatric early warning system – utilisation and mortality avoidance– the PUMA study protocol

**DOI:** 10.1186/s12887-018-1210-z

**Published:** 2018-07-25

**Authors:** Emma Thomas-Jones, Amy Lloyd, Damian Roland, Gerri Sefton, Lyvonne Tume, Kerry Hood, Chao Huang, Dawn Edwards, Alison Oliver, Richard Skone, David Lacy, Ian Sinha, Jenny Preston, Brendan Mason, Nina Jacob, Robert Trubey, Heather Strange, Yvonne Moriarty, Aimee Grant, Davina Allen, Colin Powell

**Affiliations:** 1 Centre for Trials Research, College of Biomedical and Life Sciences, Cardiff University, 7th Floor Neuadd Meirionnydd, Cardiff, CF14 4YS UK; 2 School of Healthcare Sciences, Cardiff University, East Gate House, 35-43 Newport Road, Cardiff, CF24 0AB UK; 3 Division of Population Medicine, School of Medicine, Cardiff University, Neuadd Meirionnydd, Heath Park, Cardiff, CF14 4YS UK; 4 SAPPHIRE Group, Health Sciences, Centre for Medicine, Leciester Univeristy, LE1 7RH Leicester, UK; 5 Paediatric Emergency Medicine Leicester Academic (PEMLA) Group, Children’s Emergency Department, Leicester Royal Infirmary, Leicester, LE1 5WW UK; 6 Alder Hey Children’s NHS Foundation Trust, Eaton Road, Liverpool, L14 5AB UK; 7 The University of West England, Bristol, UK; 8 Hull York Medical School, University of Hull, Hull, HU6 7RX UK; 9 Morriston Hospital, Abertawe Bro Morgannwg University Health Board, Swansea, SA6 6NL UK; 10 Noah’s Ark Children’s Hospital of Wales, Cardiff and Vale University Health Board, Heath Park, Cardiff, CF14 4XN UK; 11 Arrowe Park Hospital, Arrowe Park Road, Merseyside, Wirral CH49 5PE UK; 12 NIHR NIHR Alder Hey Clinical Research Facility, Eaton Rd, Liverpool, L12 2AP UK; 13 Swansea University Medical School, Swansea University, Grove Building, Singleton Park, Swansea, SA2 8PP UK

**Keywords:** Paediatric-early warning systems, Track-and-trigger tools, Mortality, Patient safety, And quality improvement

## Abstract

**Background:**

In hospital, staff need to routinely monitor patients to identify those who are seriously ill, so that they receive timely treatment to improve their condition. A Paediatric Early Warning System is a multi-faceted socio-technical system to detect deterioration in children, which may or may not include a track and trigger tool. It functions to monitor, detect and prompt an urgent response to signs of deterioration, with the aim of preventing morbidity and mortality. The purpose of this study is to develop an evidence-based improvement programme to optimise the effectiveness of Paediatric Early Warning Systems in different inpatient contexts, and to evaluate the feasibility and potential effectiveness of the programme in predicting deterioration and triggering timely interventions.

**Methods:**

This study will be conducted in two district and two specialist children’s hospitals. It deploys an Interrupted Time Series (ITS) design in conjunction with ethnographic cases studies with embedded process evaluation. Informed by Translational Mobilisation Theory and Normalisation Process Theory, the study is underpinned by a functions based approach to improvement. Workstream (1) will develop an evidence-based improvement programme to optimise Paediatric Early Warning System based on systematic reviews. Workstream (2) consists of observation and recording outcomes in current practice in the four sites, implementation of the improvement programme and concurrent process evaluation, and evaluation of the impact of the programme. Outcomes will be mortality and critical events, unplanned admission to Paediatric Intensive Care (PICU) or Paediatric High Dependency Unit (PHDU), cardiac arrest, respiratory arrest, medical emergencies requiring immediate assistance, reviews by PICU staff, and critical deterioration, with qualitative evidence of the impact of the intervention on Paediatric Early Warning System and learning from the implementation process.

**Discussion:**

This paper presents the background, rationale and design for this mixed methods study. This will be the most comprehensive study of Paediatric Early Warning Systems and the first to deploy a functions-based approach to improvement in the UK with the aim to improve paediatric patient safety and reduce mortality. Our findings will inform recommendations about the safety processes for every hospital treating paediatric in-patients across the NHS.

**Trial registration:**

Sponsor: Cardiff University, 30–36 Newport Road, Cardiff, CF24 0DE Sponsor ref.: SPON1362–14.

Funder: National Institute for Health Research, Health Services & Delivery Research Programme (NIHR HS&DR) Funder reference: 12/178/17.

Research Ethics Committee reference: 15/SW/0084 [13/04/2015].

PROSPERO reference: CRD42015015326 [23/01/2015].

ISRCTN: 94228292 10.1186/ISRCTN94228292 [date of application 13/05/2015; date of registration: 18/08/2015]. Prospective registration prior to data collection and participant consent commencing in September 2014.

## Background

The UK paediatric mortality rate is the highest in Europe [1]. There is evidence suggesting that missed deterioration [2, 3] and difference in hospital performance contribute to outcomes [4]. Research in the adult care context identified that acute in-hospital deterioration is often preceded by a period of physiological instability which, when recognised, provides an opportunity for earlier intervention, and improved outcome [5, 6].

In the adult context, the Royal College of Physicians endorsed the implementation of a National Early Warning track and trigger tool [7] to standardise the assessment of acute illness severity, predicting that 6000 lives will be saved. The NHS Litigation Authority (NHSLA) recommends that Trusts in England use a track and trigger tool to reduce harm to patients and avail of lower insurance premiums [8]. The Confidential Enquiry into Maternal and Child Health (CEMACH) deaths [2] and National Patient Safety Agency (NPSA) (now NHS NHS Commissioning Board Special Health Authority) [9] also advocate the use of a track and trigger tool as part of an early warning system. A ‘Track and Trigger tool’ (TTT) [10] consists of sequential recording and monitoring of physiological, clinical and observational data. When a certain score or trigger is reached then a clinical action should occur including, but not limited to, altered frequency of observation, senior review or more appropriate treatment or management. Tools may be paper-based or electronic and monitoring can be automated or undertaken manually by staff.

There is currently limited evidence to support TTT use in paediatrics. The variation in accepted physiological normal ranges for respiratory and heart rate and blood pressure across the age range, make it challenging to develop a standardised tool suitable for generic application for all hospitalised children. Some single site studies [11–13] reviewed the performance of individual TTTs, with preliminary data on the sensitivity of different cut-offs for physiological measurements. However, it was difficult to prove an ‘effect’ based on the outcome measures described, since the event rate of in hospital cardiac arrest or death is low. Even if agreement existed on a particular TTT, this needs to be acted upon in everyday clinical practice and there is considerable variation in the systems and processes in place through which this is achieved and which may be consequential for effectiveness.

TTTs are commonly part of wider Paediatric Early Warning System, which in turn are always part of a wider clinical and organisational context [14–16] with a singular workplace history, culture, division of labour, skill-mix, infrastructure, workload, case-mix, leadership, resources, and specialist expertise, which may be consequential for effectiveness. There is currently wide variability in use of TTTs in practice. For example, recent work has reviewed TTTs throughout the UK [17]. Out of a possible 157 in-patient units, information was obtained from 149 (95%) hospitals. 85% of units were using a TTT but there was huge variability in the tool being used and most of these were unpublished and un-validated. The current ad hoc utilisation of un-validated TTTs and variance in organisational capacity to respond to a deteriorating child may represent a serious clinical risk.

Over 700,000 children are admitted to hospital overnight in the UK annually with 8000 admitted to Paediatric Intensive Care Units (PICU) as an emergency [18]. Half of these admissions to PICU are from wards in the same hospital, suggesting that patients deteriorated acutely or had a cardiopulmonary arrest. Missed or delayed instances of deterioration identification in hospital are “failures in care” with a physiological, psychological and social cost to the child and family [19, 20]. There is significant short-term added cost to the NHS [21] from rising cost of litigation [22]. In the current national and global financial climate the NHS is under severe pressure to make yearly cost savings. For a society that values its NHS highly, this is widely recognised to be a situation that needs to be reversed. It is estimated that 1951 child deaths in the UK would need to be prevented each year to compare with the best performers in Europe [23].

The CEMACH report (2008) identified the need for all health care professionals to be able to recognise serious illness in children [2]. It noted that not only did this involve good clinical skills and awareness of limitations but also good communication. The report highlighted identifiable failures in a child’s direct care in “…just over a a quarter deaths, and potentially avoidable factors in a further 43% of deaths.” [2]. It was from this report that a recommendation for TTTs to be used in all hospitals was made. Recently the Royal College of Paediatrics and Child Health (RCPCH), National Children’s Bureau and British Association for Child and Adolescent Public Health [24] have examined data on childhood deaths and focused specifically on interventions which may have an effect through policy and practice changes. Although health care amenable deaths appear to have fallen since the CEMACH report they are still very prevalent. Data available up to early 2013 showed in 3857 completed reviews 21% of the deaths had modifiable factors [25]. Although these were not all as result of failure to recognise the deteriorating child, the scale of the problem, given the UK’s poor record on childhood mortality, is significant. The report specifically concluded:

“*It is important that measures are taken to improve recognition and management of serious illness across the health service – both primary and secondary care; community and hospital; general practice, paediatrics, and mental health*” [24]. The report noted that comparative data between countries is extremely difficult to interpret but that significant discrepancies exist in the UK compared to the rest of Europe in respect of mortality.

There is, as yet, no consensus on the utility of the currently available TTTs and there is variance in monitoring of children and young people [26], training to aid recognition and response to deterioration and mechanisms to ensure best practice. Children admitted to hospital, and their families should have the expectation of excellent care. Therefore research that aims to reduce missed deterioration and prevent avoidable mortality, as well as limiting un-necessary NHS added cost and litigation (from failure to rescue), is both relevant and timely. A recent systematic review highlighted limited evidence for the validity and utility of TTTs [27] and therefore there is an urgent national need to develop an evidence based approach to improving Paediatric Early Warning Systems in UK practice and produce guidance to inform National bodies (such as NICE, NHSLA, RCPCH, RCN) in order to improve patient safety within the NHS.

The aim of this study is to develop an evidence-based improvement programme to optimise the effectiveness of a Paediatric Early Warning System, evaluate its feasibility and potential effectiveness in improving the prediction of deterioration and triggering timely interventions, and identify factors necessary to ensure successful implementation and normalisation.

### Study design

PUMA is a prospective, mixed-methods, before and after quasi-experimental study. It aims to develop an evidence based programme to improve Paediatric Early Warning Systems, evaluate its feasibility and potential effectiveness in improving the detection of deterioration and triggering timely interventions, and identify the factors necessary to ensure successful implementation and normalisation. The study is underpinned by a functions-based approach to intervention development. In other words, “the function and process of the intervention should be standardised, not the components themselves” [28]. The study deploys an Interrupted Time Series (ITS) design in conjunction with ethnographic cases studies and embedded process evaluation.

#### Research aims

- To identify through systematic review of the literature the evidence for the core components of an effective TTT and a Paediatric Early Warning System.
- To identify the contextual factors that are consequential for TTT and Paediatric Early Warning System effectiveness.
- To develop and implement an evidence-based improvement programme to optimise the effectiveness of Paediatric Early Warning System for prospective evaluation.
- To evaluate the ability of the programme to optimise the ability of Paediatric Early Warning System to identify serious illness and reduce clinical events by examining core outcomes.
- To identify the key ingredients of successful implementation and normalisation.

Parent and young people will be involved throughout the study, a parent advisory group will be set up by a public and patient involvement manager who will train them and work with them throughout, to ensure that their views and inputs are representated. The group will focusparticularly on design of information leaflets, interview schedules, qualitative data analysis and dissemination acitivties.

**Fig. 1:**
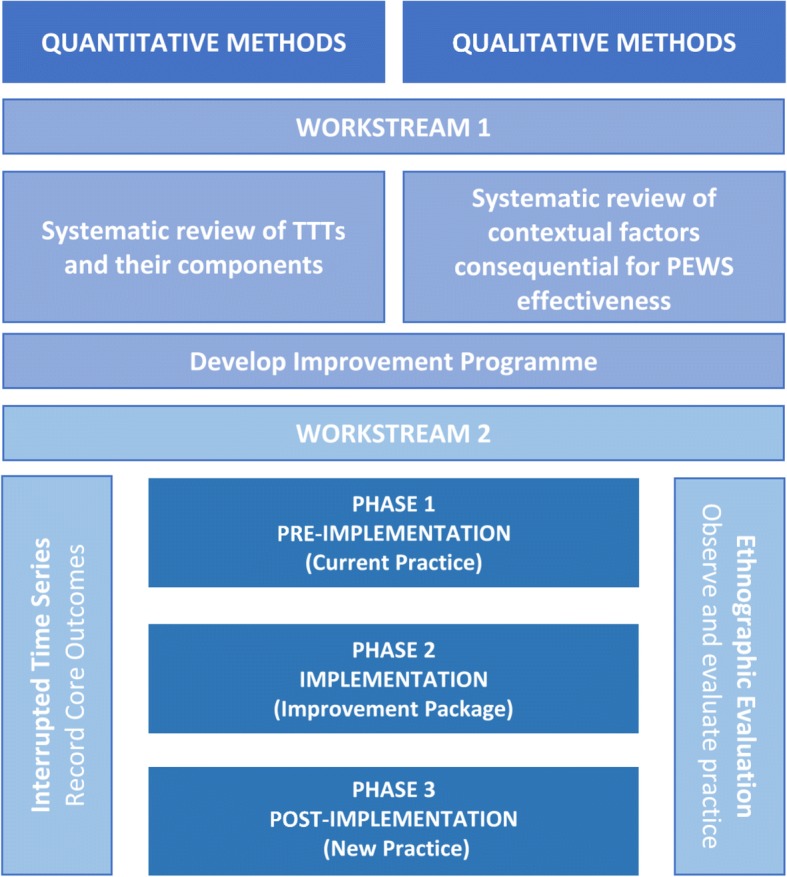
PUMA Study Design

The PUMA study is divided into two parallel workstreams (see Fig. 1):

***Workstream 1.***

The development of a programme to improve Paediatric Early Warning Systems based on systematic review (SR) and an implementation package based on effective strategies identified in the SR.

***Workstream 2.***

A prospective mixed method, before and after study design with ITS core outcome evaluation and embedded ethnographic case studies in four hospitals.

The ITS design is an effective quasi-experimental design and an alternative to the randomized controlled trial. Because it avoids the potential biases in the estimation of intervention by considering the time series factors, such as seasonal trends and autocorrelation, it is increasingly adopted in the evaluation of health care interventions, where randomised controlled trials (RCTs) are not feasible.

The effectiveness of the improvement programme in optimising the Paediatric Early Warning System will be assessed by examining the core outcomes defined in the statistical section below. The primary analysis of the outcomes will be an interrupted time series for each of the four hospitals. This aims to identify a change in the rate of outcomes that are potentially attributable to the introduction of the programme to improve the Paediatric Early Warning System. The interrupted time series is adopted here for the main quantitative analysis.

The embedded ethnographic case studies conducted within each phase of the study will evaluate usual Paediatric Early Warning System practice, the process of implementation, and the impact of the improvement programme on the Paediatric Early Warning System post implementation.

The plan for the second workstream is divided into three phases:

Phase 1) Observe and record outcomes in current practice.

Phase 2) Implement the programme to improve Paediatric Early Warning Systems and undertake a concurrent implementation process evaluation.

Phase 3) Evaluate the impact of the improvement programme on the Paediatric Early Warning System.

#### Theoretical framework

Healthcare improvement initiatives involve introducing interventions into complex social-technical systems. The dynamic nature of different technical, social, institutional and political factors affects the mechanisms by which an intervention has its effect. Interventions are ‘actors’ or ‘events’ within a system [29, 30], which afford or constrain healthcare processes, require particular preconditions to work effectively, and interact with other technologies, people and processes [30]. In simple terms, the way in which an intervention interacts with each context affects its function.

Thus, in developing, evaluating and implementing any intervention it is important to take into account relevant contextual features in order to establish the local modifications necessary to ensure sustainability and success, and to do this it is essential that an intervention’s generative mechanisms (its functions) are understood and can be articulated.

The study design is therefore informed by the premise that implementing interventions in real world settings requires consistency in process and function, rather than form [28]. Instead of standardising intervention components (e.g. a TTT, education workshops, a handover tool for nurses and doctors), standardisation should occur in the change process or key functions that these components aim to achieve. For example, “a handover tool for nurses and doctors” is better regarded as a mechanism to ensure key patient information is communicated between professionals. This mechanism could then take on different forms according to local context, while still achieving the same goal.

In addition, in order to think systematically about improving Paediatric Early Warning Systems and the socio-technical contexts into which an improvement programme will be introduced, we will deploy Translational Mobilisation Theory (TMT) [30, 31]. TMT is a practice theory which builds on ecological approaches to work, activity theory and Actor Network Theory to describe projects of goal-oriented collective action in conditions of emergence and complexity. ‘Projects’ are the basic unit of analysis in TMT and refer to an institutionally sanctioned socio-material network of time-bounded cooperative action and actors that follows a trajectory in time and space: in this case the detection of physiological deterioration and timely intervention in the care of sick children. Projects are given their form by Strategic Action Fields (SAF) which generate the institutional contexts in which projects are progressed and which provide the socio-material resources for collective action. The importance of understanding context for quality improvement purposes is well established. TMT provides a framework to support systematic attention to the salient features that condition projects of social action and which are likely to be consequential for the success or failure of an intervention. TMT directs attention to the mechanisms through which projects of collective action are mobilised - object formation (how actors create the objects of their practice), reflexive monitoring (practices through which actors evaluate a field of action to generate awareness of project trajectories), articulation work (practices that assemble and align the diverse elements through which object trajectories and projects of collective action are mobilised), translation (practices that enable practice objects to be shared and differing viewpoints, local contingencies, and multiple interests to be accommodated in order to enable concerted action), sense-making (practices though which actors order, construct, and mobilise projects and enact structures and institutions).

Normalisation process theory (NPT), which has a high degree of conceptual affinity with this underlying theoretical framework, will provide an additional theoretical lens to inform tool implementation and the evaluation of this process. NPT is concerned with ‘how and why things become, or don’t become, routine and normal components of everyday work’ [32] and it defines four mechanisms that shape the social processes of implementation, embedding and integrating ensembles of social practices. These are interrelated and dynamic domains and include: ‘coherence’ (the extent to which an intervention is understood as meaningful, achievable and desirable); ‘cognitive participation’ (the enrolment of those actors necessary to deliver the intervention, which, for our purposes can be human and non-human); ‘collective action’ (the work that brings the intervention into use); and ‘reflexive monitoring’ (the ongoing process of adjusting the intervention to keep it in place). We will use these domains as a framework to analyse the contextual factors necessary for integration into routine work organisation (normalisation). NPT and TMT are relatively new theories and we will be open to the possibility of contributing to their refinement in the light of our findings.

### Setting

The recent survey of paediatric units in the UK reported that 90% of tertiary units and 83% of District General Hospitals (DGH’s) already had a tack and trigger tool in place. A convenience sample of paediatric units was selected for the study to represent types of unit and those with and without a TTT in place. These four hospitals represent paediatric inpatient units of varying size; two specialist children’s hospitals with PICUs and two large DGHs (see Table 1). No studies so far have involved a DGH environment. It is important if a Paediatric Early Warning System is going to be used throughout UK we can capture this environment, where most children are admitted.

**Table 1:** Characteristics of participating hospitals

Site	Hospital type	Number of beds (excluding PICU)	Approximate number of in-patient admissions annually (excluding day-cases)	TTT currently in place?
Site 1	Specialist children’s hospital	337 in-patient, 15 HDU	Over 200,000	Yes
Site 2	District General hospital	22 in-patient, 2 HDU	2500	Yes
Site 3	Specialist children’s hospital	116 in-patient, 6 HDU	23,000	No
Site 4	District General hospital	38 in-patient, 7 HDU	7500	No

## Study procedures and methods

### Workstream 1: Evidence review of TTTs and socio-material and contextual features of successful Paediatric early warning systems, and development of functions-based improvement programme

#### Objectives

- Identify through a systematic review of the literature the evidence for the core components of a paediatric TTT.
- Identify through systematic review of the literature the evidence for the core components of a Paediatric Early Warning System.
- To identify the contextual factors that are consequential for TTT and Paediatric Early Warning System effectiveness.
- Develop theories about the mechanisms by which the core components of Paediatric Early Warning Systems have their effects.
- Develop a evidence-based improvement programme to optimise the effectiveness of Paediatric Early Warning System for use in different contexts.

A systematic review will be conducted in order to answer three interlinked questions:

**Q1. How well validated are existing TTTs for Paediatric Early Warning Systems and their component parts?**

We will identify studies which have developed and/or validated TTTs (or core items). These will allow us to identify a set of best items for a tool and to guide trigger points for a tool.

**Q2. How effective are Paediatric Early Warning Systems (with or without TTT) at reducing mortality and critical events?**

We will identify RCTs and quasi-experimental studies which have evaluated Paediatric Early Warning Systems (with or without TTTs). These will allow us to identify the potential components of a successful Paediatric Early Warning System.

**Q3. What socio-technical and contextual factors are associated with successful or unsuccessful Paediatric Early Warning System (with or without TTT)?**

We will utilise studies included in Q1 and Q2 where relevant information on implementation factors are included and also qualitative or quantitative studies of Paediatric Early Warning System implementation. This will allow us to develop programme theories for the core components and mechanisms of Paediatric Early Warning Systems and identify factors consequential for implementation and normalisation. If there are gaps in the literature relating to paediatrics then this area may be extended to consider factors in adult implementation and other related literatures.

#### Search methods

The Cardiff University Support Unit for Research Evidence (SURE) will undertake the searches (http://www.cardiff.ac.uk/insrv/libraries/sure/index.html). Our review is registered with the PROSPERO database [33]. A comprehensive search will be conducted across a range of databases from the study’s inception to identify relevant evidence/studies in the English language. Published literature, including studies in press, will be considered. To identify published resources that have not yet been catalogued in the electronic databases, recent editions of key journals will be hand-searched.

##### Identify relevant studies

The search results will be imported into the reference management database Endnote. Duplicate references and clearly irrelevant citations will be removed. All remaining studies will then be sent to reviewers to screen for relevance and categorized according to which line of analysis they contribute to. All identified titles and abstracts will be reviewed by two reviewers for inclusion and also which of the three questions they could contribute to. Studies considered potentially relevant by either reviewer will be retrieved in full. Full texts will be reviewed in full by two reviewers against the eligibility criteria and classification as to which questions they contribute to be re-assessed. Disagreement between reviewers will be resolved by consensus in the group, with reasons for exclusion recorded.

##### Data extraction

The data extraction form will have some common elements (study design, country, setting, exact population, nature of the Paediatric Early Warning System, outcomes assessed), then specific sections for each of the three questions. Data to be extracted:

- Q1 – items in the TTT, predictive ability of individual items and overall combination, sensitivity and specificity, inter and intra-rater reliability
- Q2 – critical events, morbidity, mortality
- Q3 – socio-technical features associated with successful and unsuccessful Paediatric Early Warning Systems, factors consequential for implementation and normalisation

The question specific information will be extracted by members of the team focussed on that question.

##### Risk of Bias assessment

Studies will be quality appraised according to the purposes for which they will be used. For Q1 and Q2 we will utilise appropriate quality appraisal tools according to study type using the checklist suggested by Downs and Black [34]. However, Q3 is concerned with theory generation, here it is evidential fragments or partial lines of inquiry rather than entire studies that form the unit of analysis. In such cases, the quality of each item will be appraised according to the contribution it makes to the developing analysis.

##### Data synthesis

Q1 will combine information using the median ROC (if data is available) to identify the quality of prediction. The potential range of predictions of each item will be tabulated and associations between each item and the outcome will be summarised using odds ratios (OR) and 95% confidence intervals.

Q2 will use a random effect meta-analysis of the OR of mortality or critical event in the intervention group compared to control.

Q3 will involve a theory driven and theory generating qualitative synthesis of Paediatric Early Warning System active ingredients, evidence of the mechanisms by which they have their effects in different contexts, and factors associated with implementation and normalisation in order to develop an indicative programme theory.

Drawing on the evidence from the literature review, we will devise a theoretical model of an optimal Paediatric Early Warning System. We will identify the core functions of the system and develop an improvement programme, including implementation resources. Each of the four centres will have a local PI acting as a champion for the implementation. Each champion, along with members of their ‘improvement team’ will be required to attend a briefing session, facilitate assessment of their system to identify opportunities for improvement, attend an action planning session to identify potential solutions, and use resources provided in the implementation guide to facilitate implementation.

#### Outputs from Workstream 1

1. Systematic review of paediatric TTT development and validation.
2. Systematic review of paediatric TTT effectiveness.
3. A qualitative narrative review of Paediatric Early Warning Systems in different contexts.
4. The development of theories about the core functions of effective Paediatric Early Warning Systems and how these can be implemented in different contexts, and the factors consequential for implementation and normalisation.
5. Paediatric Early Warning System improvement programme for both DGHs and specialist children’s hospitals.

### Workstream 2: Prospective before and after evaluation with embedded case studies

#### Objectives

- Evaluate the ability of the Paediatric Early Warning System improvement programme to impact on clinical outcomes.
- Identify the contextual factors that are consequential for Paediatric Early Warning System effectiveness.
- Develop evidence-based recommendations for a national Paediatric Early Warning System improvement programme with underpinning programme theories.
- Identify the key ingredients of successful implementation and normalisation.

#### Time interrupted series (ITS) analysis – To evaluate core outcomes

This before and after study evaluation will be conducted in three phases:

***Phase 1:***

The baseline phase will be conducted to observe current practice and establish the foundations for the interrupted time series (ITS) analysis of the outcomes including mortality and the critical events listed in detail in the statistical considerations section.

This phase will last 12 months for all four hospitals. A 12 month period has been chosen to give a reasonable number of data points (months) for the time series and to accommodate for seasonal differences in case mix.

***Phase 2:***

The implementation phase within each hospital will take up to 12 months. This will involve working with hospital management and multidisciplinary staff to implement and embed improvements to the Paediatric Early Warning System. Outcome data will continue to be collected during this phase to give an uninterrupted time series.

***Phase 3:***

The post implementation phase will focus on the impact of improvement to the Paediatric Early Warning System on outcomes and will last a further 12 months to give an appropriate number of data points (months) for the time series and to accommodate for seasonal differences in case mix. Outcome data will be collected, which should also now include the TTT (where measured).

Overall for each hospital the study will last for 36 months, the intervention will occur concurrently in each of the four hospitals. We will collect audit data on mortality and specified morbidity (rates per 1000 non-ICU patient-days) before during and after implementation and fit a time series (36 time points) per hospital and test for changes in slope associated with time intervention. This will enable us to estimate the effect of the improvement progrmamme on mortality and significant morbidity.

#### Embedded case studies: To explore current practice, revised practice and response to the improvement programme

Organisational case studies will be undertaken in one ward within each hospital. Ethnographic methods, (non-participant observation and interviews), will be deployed to explore the technical, social, and organisational factors consequential for Paediatric Early Warning System effectiveness. In each case we will undertake a pre and post implementation review of the local Paediatric Early Warning Systems in the clinical settings prior to, and after implementation of the improvement programme, to assess the impact on practice (see Table 2 for a summary of workstream 2).

**Table 2:** Summary of workstream 2

Data collection phase	Aim	Purpose	Approach
PHASE 1: Pre-Implementation	To understand current practice.	To identify the micro, meso and macro contextual features consequential for effectiveness of an improvement programme.	Non-participant observation of everyday practice (*n* = 250 h) Interviews with staff & service managers (n = 48) Interviews with parents (*n* = 32)
PHASE 2: Implementation	To develop an improvement strategy tailored to each organisation.	Guided by the systematic review, we will identify factors that appear to support the normalisation of changes to the Paediatric Early Warning Systems in practice and will draw on these materials to inform our improvement programme.	Process evaluation with two elements; Observational methods to describe and understand the impact of key elements of the improvement programme. A range of methods including interviews and observations to explore experiences of, and responses to, the system changes.
PHASE 3: Post-implementation	To understand the impact of the Paediatric Early Warning System improvement programme on practice.	To explore in detail staff experiences of the Paediatric Early Warning System improvement programme, factors consequential for impact, and any unintended consequences.	Non-participant observation of everyday practice (*n* = 150 h) Interviews with staff & service managers (n = 48) Interviews with parents (*n* = 32)

Data will be generated through ethnographic fieldnotes recorded in relation to: non-participant observation of everyday practice (by shadowing individuals – nurses, doctors, support staff), attendance at, and where possible digital recording of, key meetings and events, interviews with clinical team members, service managers and parents, and the analysis of relevant documents.

Our concern will be with understanding the network of actors: people, processes, technologies and artefacts, and their interrelationships in each Paediatric Early Warning System. Drawing on our theoretical framework, the literature review, we will develop a template to guide our observations and interviews. Data generation will not be absolutely constrained by this however; rather in each case the strategy will be to ‘follow the actors’ (human and non-human). This will ensure that there is a consistent approach across case studies to facilitate comparative analyses, but flexibility to modify data generation in response to the singular features of each site. We will focus on what participants do, the tools they use, the concepts they deploy, and consider what these practices reveal about what they know and the factors that facilitate and constrain action [35]. Adopting a TMT lens will direct attention to the socio-material relationships within each Paediatric Early Warning System and the impact of the local institutional context in conditioning the possibilities for action [31, 36].

Observations will be undertaken over a period of up to six weeks in each case, in order to give sites sufficient time to become accustomed to having a researcher in their midst, and so we can develop an accurate understanding of normal practice. Observations will be conducted at different times of day/night and on different days of the week, including weekends, to ensure a range of time periods are covered.

In addition we will also undertake 6–8 interviews with parents/carers to explore their views and experiences (*n* = 32) and semi-structured digitally-recorded interviews with a sample of clinical staff and relevant service managers (*n* = 48). Audio recordings will be transcribed verbatim and analysed to explore each Paediatric Early Warning System at micro, meso and macro levels. The aim will be to develop a clear description and understanding of the local Paediatric Early Warning Systems in each case.

Observations will be recorded contemporaneously as low inference-style field notes and expanded on as soon as practical after the data was collected. Interviews will be digitally recorded with consent, and will be organised to take place either in private offices or by telephone. Interviews with a purposively selected sample of parents who have a physiologically unstable child will be undertaken when the child is still an in-patient, but at a time when their condition is considered by clinical staff to be stable. For the purposes of this study we will not include parents whose child has died but will interview parents whose (a) child has been monitored only (b) received intervention to prevent deterioration (c) had a critical event. Documents/records will be treated as both a resource and a topic. Their content will be analysed to inform our understanding of organisational processes and practices. Their form will be analysed in order to develop a better understanding of their design and affordances and inter-relationships.

We will replicate this ethnographic process (both non-participant observations and interviews) following implementation of the programme, modifying the interview style and content, as well as the primary focus of the observations, in order to explore in detail staff experiences of the system, factors consequential for impact, and any unintended consequences. We will also reassess the Paediatric Early Warning System using the structured template as a guide to observation, in order to analyse changes in these relationships brought about by the improvement programme, and the implications this has for normalisation.

#### Paediatric early warning system improvement and evaluation

An improvement strategy will be tailored to each organisation. Each of the four centres will have a local Principal Investigator (PI) acting as a study champion for the implementation of the improvement programme. The systematic review will be used to identify those factors that appear to support the normalisation of changes to the Paediatric Early Warning Systems in practice and we will draw on these materials to inform our improvement programme. The process evaluation has two elements: (i) evaluation of the implementation of the improvement programme to site PIs; (ii) the local implementation of Paediatric Early Warning System improvements.

##### Implementation of the improvement programme

Observational methods will be employed to describe and understand the impact of key elements of the improvement programme, including a briefing and action planning session with tailored facilitation via fortnightly calls with the site champions throughout the implementation phase. Observations will focus on the content of the programme components and also how they are delivered by members of the PUMA research team to local champions at each of the four study sites. Data will be audio-recorded and transcribed, and observers will also take low inference style field notes, which will be later word-processed.

##### Local system improvements

Various methods, including interviews and observations, will be employed throughout the implementation phase to explore experiences of, and responses to, the system changes implemented as part of the improvement programme. Observers will record barriers and facilitators (clinical, management and organisational) to implementation in local contexts and plans for how these are to be overcome. Interviews will be conducted with PIs at the end of the implementation phase, either by phone or face-to-face. In addition, for each hospital we will evaluate service level implementation through interviews with a selection of staff to explore their experiences, and views of the improvement programme (approx. *n* = 40). Interviews will be arranged to fit around the clinical responsibilities of service providers and can be undertaken either face to face or by telephone. This will be undertaken after the implementation phase of the study in order not to unduly influence the implementation process.

### Statistical considerations

#### Primary outcome measure

The primary outcome measure is a composite outcome, measuring the number of children who experience at least one of the following events each month, per 1000 patient bed days:

- Mortality
- Cardiac arrest
- Respiratory arrest
- Unplanned admission to PICU
- Unplanned admission to PHDU

#### Secondary outcome measures

The secondary outcome measures are single outcome measures, where we look at the monthly rates of the following critical events separately, per 1000 patient bed days:

- mortality
- unplanned admission to PICU
- unplanned admission to PHDU
- cardiac arrest
- respiratory arrest
- medical emergencies requiring immediate assistance
- reviews by PICU staff
- Critical Deterioration metric [37] or equivalent measure
- PIM3 at PICU admission

#### Sample size

A simulation-based approach [38] to calculate the power has been used as it is challenging to derive a formula for the sample size [39]. With the event rate of unplanned admission to PICU (206/20696 = 1%) and the monthly admission to hospital overnight from historical data from two of our sites (one tertiary one DGH), we obtained the monthly prevalence of unplanned admission to PICU at pre-intervention stage. Tibbals [40] have shown that implementation of calling criteria (similar to a track and trigger tool) with a rapid response team resulted in a risk ratio of 0.65 in terms of total avoidable hospital mortality. We assumed that the implementation of the new intervention package will result in a similar risk ratio. For comparing the pre- and post- intervention monthly events, this results in a potential the effect size of 2.8 with mean difference 2.0 and common standard deviation 0.7. With effect size at least 2.0 [38], a total of 24-month observations (12-month pre-intervention phase and 12-month post-intervention phase) would give this study 90% power. Given the potential for seasonal effects, we have taken this as a conservative approach for the sample size.

### Analysis

#### Quantitative analysis

##### Main analysis

Each hospital will be regarded as a separate interrupted time series and the autoregressive integrated moving average (ARIMA) [41] model will be used for the analysis. This aims to identify a change in the monthly rate of mortality and the following critical events; unplanned admission to PICU or PHDU, cardiac arrest, respiratory arrest, medical emergencies requiring immediate assistance (arrest calls who were not respiratory or cardiac arrests), reviews by PICU staff and critical deterioration [42]. First-order autocorrelation will be tested by using the Durbin-Watson statistic, and higher-order autocorrelations will be investigated by using the autocorrelation and partial autocorrelation function. As some hospitals will switch from paper-based systems to electronic-based systems, this factor will be added in the models accordingly to accommodate the impact of the change. The changes of level and of slope at the adjacent time point between pre-implementation and post-implementation phases will be analysed and we will conclude the effectiveness of the intervention if either of these two changes is statistically significant at a 5% level [43].

##### Secondary analysis

As low/zero monthly rates may occur in critical events (such as mortality), we will monitor the measures of these outcomes and consider alternative time series approach for the analysis of those with non-ignorable zero values.

We will adapt the Critical Deterioration (CD) metric originally defined by Bonafide and colleagues as an unplanned transfer to an intensive care unit followed by non-invasive or invasive mechanical ventilation or vasopressor infusion within 12 h [37]. In the Paediatric Intensive Care Audit Network (PICANET) database, the relevant information for this outcome is collected in calendar days. Therefore, we will report equivalent critical interventions that occur within the first one or two calendar days of admission and provide the figures for comparison. Where there are cases of incomplete patient bed days we will impute by the average patient bed days of that month and the then compare the adjusted figures with the original ones as a sensitivity analysis. We will utilise the PICANET data to re-calculate the unplanned PICU admission and compare the figures with what we collected from lcoal hospitals as a sensitivity analysis.

We will compare the severity of illness in children admitted to PICU using PIM3, which is a model to assess the child’s risk of mortality among children admitted to PICU. This information is collected for all children admitted to PICU in the PICANET database.

#### Qualitative analysis

For each phase (pre-implementation, implementation and post-implementation) data generation and analysis will be undertaken concurrently, facilitating a progressive narrowing of focus designed to develop in-depth understanding of the Paediatric Early Warning Systems, the improvement programme and implementation process in each case and the implications of the improvement programme for practice. The various materials collected (field notes, interview transcripts, documents) will be used in a triangulating fashion to develop concrete descriptions of relevant aspects of Paediatric Early Warning Systems targeting the key themes and topics of specific analytic concern. Parent and patient representatives will contribute to this process.

Analysis will be undertaken in four stages.

Stage 1 will develop a description and analysis of the Paediatric Early Warning System in each case: people, processes, structures, technologies and artefacts and their interrelationships.

Stage 2 analysis will concentrate on processes to implement the improvement programme in each case. We will explore the ‘coherence’ of the improvement programme to optimise Paediatric Early Warning Systems from the perspective of participants, participant’s experiences of the of the programme in enrolling actors (human and non-human) necessary for implementation and the reasons for this; the work necessary to bring improvements into use; and the ‘reflexive monitoring’ necessary to keep these in place.

Stage 3 will evaluate Paediatric Early Warning Systems post implementation of the improvement programme in each case. As in stage 1 we will develop a description and analysis of the Paediatric Early Warning System: people, processes technologies and artefacts and their interrelationships. We will assess the changes that have taken place pre and post improvement programme and the normalisation of these using the four domains of NPT to inform our analyses.

Stage 4 will triangulate all data to build up a picture of improvement programme to optimise Paediatric Early Warning Systems, and the factors consequential for its pattern of impact. Within and cross-case analysis will be undertaken to develop an analysis of the relationship between the programme, context, mechanisms and outcomes in order to inform the implementation of a national improvement programme to optimise Paediatric Early Warning Systems.

## Discussion

This paper presents the background, rationale and design for this mixed methods study examining the evidence for an evidenced based programme to improve Paediatric Early Warning Systems with evaluation of its potential effectiveness. This will be the most comprehensive study of Paediatric Early Warning Systems in the UK, and the first to be underpinned by a functions-based approach to intervention development, with the aim to improve paediatric patient safety and reduce mortality. Our findings will inform recommendations about safety processes that should be established in every hospital treating paediatric in-patients across the NHS.

### Limitations

Mortality is fortunately an infrequent outcome in acute paediatrics in the UK. This study may not have sufficient numbers of patients to be able to show the impact on mortality of a systems approach to improve patients’ safety. There will be a large number of secondary outcomes both qualitatively and quantitatively, which are likely to show change over time. The functions-based Paediatric Early Warning System programme is new and a radical challenge to orthodox approaches to improvement, which typically focus on the implementation of discrete interventions. Furthermore, implementing a system-wide programme of improvement is likely to be challenging in a cost constrained NHS. It will be important that any recommendations are practical and feasible. An assessment of the feasibility of such an approach will be a key outcome of the study.

### Time line

The study commenced in September 2014 and the end of the third phase of the study will finish in April 2019.

## Abbreviations

CD: Critical Deterioration
CEMACH: Confidential Enquiry into Maternal and Child Health
DGH: District General Hospital
HDU: High Dependancy Unit
ITS: Interrupted Time Series
NHS: National Health Service
NHSLA: NHS Litigation Authority
NICE: National Institute for Clinical Excellence
NPSA: National Patient Safety Agency
NPT: Normalisation Process Theory
PI: Princiapl Investigator
PICANET: Paediatric Intensive Care Audit Network
PICU: Paediatric Intensive Care Unit
RCN: Royal College of Nursing
RCPCH: Royal College of Paediatrics and Child Health
TMT: Translational Mobilisation Theory
TTT: Track and Trigger Tool
